# 48-week efficacy and safety of transitioning virologically stable HIV-1 patients from nevirapine IR 200 mg BID to nevirapine XR 400 mg QD (TRANxITION)

**DOI:** 10.1186/1758-2652-13-S4-P45

**Published:** 2010-11-08

**Authors:** K Arastéh, A Ward, A Plettenberg, JM Livrozet, C Cordes, A Winston, E Wang, A Quinson

**Affiliations:** 1 EPIMED, Vivantes Auguste-Viktoria-Klinikum, Berlin, Germany; 2 Dupont Circle Physicians Group, Washington DC, USA; 3 Asklepios Klinik St. Georg, Hamburg, Germany; 4 Hôpital Edouard Herriot, Lyon, France;; 5 St Mary's Hospital, Imperial College, London, UK; 6 Boehringer Ingelheim Inc, Ridgefield Connecticut, USA

## Background

Wk 24 TRANxITION study data showed patients transitioned from immediate release nevirapine (NVP IR) twice daily (BID) to NVP extended release (NVP XR) once-daily (QD) demonstrated non-inferior efficacy to patients continuing on IR NVP BID [1]. Similar safety was reported for NVP XR and NVP IR in the VERxVE study [2]^2^. Wk 48 efficacy/safety data from TRANxITION study are presented here.

## Methods

Open label, randomized (2:1), non-inferiority, parallel group study comparing NVP XR 400 mg QD with NVP IR 200 mg BID in HIV-1 patients >18 years receiving IR NVP plus one of three NRTI combinations, with viral load (VL) <50 copies/mL. Patients remained on their previous background therapy for treatment duration. Sustained virologic response (VL <50 copies/mL) was assessed at Wk 48 using a time-to-loss of virologic response (TLOVR) algorithm.

## Results

426 patients completed 48 wks of treatment. 94.9% of NVP XR and 91.9% of NVP IR patients. Mean baseline CD4+ counts: 557.7 cells/mm^3^ and 569.7 cells/mm^3^, respectively. 48 Wk data are reported in Table 1. Non-inferiority of virologic suppression was achieved using a TLOVR and snapshot analysis.

**Table 1:** 

Parameter	NVP XR QD (N=295)	IR NVP BID (N=148)	Difference (95% CI)
Virologic response (VL <50 copies/mL, TLOVR-FAS), n (%)	261 (88.5)	130 (87.8)	0.6 (-5.9, 7.1)*

CD4+ count cells/mm3 (LOCF), mean (SD)	52.1 (140.5)	81.6 (138.2)	-

AEs, n (%)	255 (86.4%)	108 (73.0%)	-

DAIDS Grade 3-4	19 (6.4%)	9 (6.1%)	-

SAEs, n (%)†	30 (10.2%)	12 (8.1%)	-

*Based on Cochran's statistic †None drug related, FAS = full analysis set; AE = adverse event; SAE = Serious adverse event

AEs were mostly mild-moderate in both groups with a higher reported rate of gastrointestinal AEs in XR. The proportion of patients with DAIDS Grade 3/4 AEs was similar in the XR and IR groups.

## Conclusions

At Wk 48, non-inferiority between the NVP XR 400 mg QD and NVP IR 200 mg BID groups was sustained. No unexpected AEs were observed at Wk 48. These data support transition from NVP IR to NVP XR in patients stable on the former formulation.
